# Do we interpret ambiguity and feel according to how we define ourselves? Relationships between self-perception, interpretation biases, and their role on emotional symptoms

**DOI:** 10.3389/fpsyt.2024.1502130

**Published:** 2024-12-20

**Authors:** Oscar Martin-Garcia, Ivan Blanco, Alvaro Sanchez-Lopez

**Affiliations:** Department of Personality, Evaluation and Clinical Psychology, Complutense University of Madrid, Madrid, Spain

**Keywords:** cognitive latent schema, self-discrepancies, negative interpretation bias, emotional symptoms, longitudinal design

## Abstract

**Introduction:**

In today's fast-paced world, depression and anxiety are the most prevalent health problems, generating high economic and social burdens. Interpretation biases seem to play a pivotal role in this emotional problems, influencing how individuals interpret emotionally ambiguous information. These interpretation biases can emerge due to the activation of latent schemas related to how individuals perceive themselves. Therefore, integrating the study of cognitive and self-discrepancy models can offer a comprehensive approach to better understanding the onset or maintenance of emotional symptoms, through their relationship with interpretation biases. In this paper, we aimed to test whether differences in self-perception might act like a cognitive schema that activate cognitive bias, influencing information processing and predicting emotional symptoms.

**Method:**

Seventy-three undergraduates completed two different experimental tasks, evaluating self-discrepancies and self-referential negative interpretation bias. Moreover, emotional symptoms were collected after completing the tasks and 1-2 months after, prior to coping with a natural stressor (exam period). The main analyses comprised mediational models, both cross-sectional and longitudinal, with the aim to test whether interpretation bias might act like a mediator in the relation between self-discrepancies and emotional symptoms.

**Results:**

First, the results showed significant correlations between higher levels of self-discrepancies (actual-ideal and actual-ought) and higher levels of emotional symptoms (depression and anxiety), as well as with higher negative interpretation biases. Further, cross-sectional mediational models showed that negative interpretation biases partially mediated the relationship between self-discrepancies and emotional symptoms. As for the longitudinal mediation analysis, interpretation bias only mediated the specific relation between actual-ideal self-discrepancies and increases in anxiety symptoms, while the rest of the indirect effects were not significant.

**Discussion:**

These results suggest that self-discrepancies could be understood as indices of the activation of latent cognitive schemas, which would influence subsequent stages of information processing, such as negative interpretations of ambiguous information, partly accounting for the emergence and/or maintenance of emotional symptoms.

## 1 Introduction

In the dynamic and often overwhelming pace of modern life, mental health has emerged as a global priority, with depression and anxiety being the most highly prevalent psychological problems. As some studies report, the prevalence of these emotional disorders is very high. The lifetime prevalence of depression symptomatology ranges between 2-21% (1) and near a 4% of the population suffers from anxiety disorders (2). This tendency has been increasing over the decades (2, 3). These conditions are not merely individual problems but represent collective challenges that deeply affect our society in multiple dimensions, implicating socioeconomic consequences. At the individual level, these conditions can lead to a significant decrease in quality of life, interfere with the ability to maintain healthy personal and professional relationships, and in some cases, even lead to suicidal ideation and suicidal attempts (4–6). Further, mental health disorders are associated with economic losses due to lost productivity and healthcare costs (7–9). This context highlights the need for better understanding the mechanisms that can influence the appearance and maintenance of emotional problems, generating new models that permit to improve the prediction of their course and risk of relapses.

From a cognitive perspective, cognitive biases have been conceptualized as key mechanisms for the emergence of emotional symptomatology (10–14). From a traditional point of view, these biases are understood as tendencies to preferentially processing some types of emotional information over other ones, giving rise to differences in emotional and behavioral aspects as a consequence of this biased processing (14). One of the most studied biases is the interpretation bias, referred to the individual’s tendency to interpret ambiguous information in more negative or positive ways. For example, in a context or scenario where a boss does not greet his/her workers when he/she always use to do it, some individuals may generate a negative interpretation centered on interpreting that his/her boss is unhappy with their job, while others individuals may interpret this situation on a more neutral way, centered on the fact that his/her boss slept badly the last night. Several studies have assessed the presence of negative interpretation biases in different emotional problems, such as depression (15) and anxiety (16), showing the existence of differences between people with and without emotional symptoms, namely negative interpretation biases being higher in individuals with higher depression and anxiety levels (17–21). Consequently, negative interpretation biases seem to be relevant to understanding emotional dynamics, making it important to clarify their functioning, and factors involved in their occurrence.

Following Beck’s traditional model [for later reviews, see (22)], these biases would arise as a consequence of the interaction between a series of latent negative schemas in the individual and the occurrence of contextual stressors. Latent schemas refer to representations of stimuli, ideas or experiences that are internally stored in memory, giving rise to core internal beliefs that guide the interpretation of the experienced situations (e.g., “if I do not get to achieve everything that I have set out to achieve, I am a loser”). According to this framework, when a schema is triggered by a stressor, the meaning of the experienced situations is interpreted based on this schema, influencing all the subsequent cognitive, emotional, motivational, and behavioral processes. These latent negative schemas might have different contents. For example, the abovementioned Beck’s cognitive model proposed the existence of different nuclear schemas pertaining to beliefs about the world, the others, and oneself (i.e., self-referential schemas). In this sense, the activation of these self-referential schemas might influence the subsequent processes related to how an individual defines him/herself, which is understood as self-perception. Self-perception is, however, also defined by other specific self-domains. For example, the Self-Discrepancy Theory [SDT: (23–25)], postulates the existence of different self-domains, namely, the actual-self (i.e., attributes that an individual truly believes they possess), ideal-self (i.e., attributes one ideally wants to possess) and the ought-self (i.e., attributes one believes they should possess). It is the discrepancies among these self-domains that have consistently been associated to different emotional problems. Concisely the SDT pinpoints actual versus ideal-self (i.e., actual-ideal) discrepancies to be related to higher levels of depressive symptoms and actual versus ought-self (i.e., actual-ought) discrepancies to be related to higher levels of anxiety symptoms (23–26).

Thus, it is the discrepancies between the different self-domains that offer a closer look at the processes related to self-perception. Given that self-referential schemas cannot be directly measured, assessing indices of self-discrepancies could offer an indirect evaluation of Beck’s former schemas. In fact, this idea has been considered not only in theoretical models [e.g., (23–26)], but also in experimental studies [e.g., (27)]. For example, some studies have shown the activation of certain latent schemas through priming effects of different self-discrepancies (27). These studies seek to establish the differentiating role of specific self-discrepancies in the emergence of specific emotions, in order to establish whether their activation can affect the emotional state of the individual. For instance, in the study of Strauman and Higgins (27), participants were differentiated according to their levels of self-discrepancies (i.e., high actual-ideal self-discrepancies and high actual-ought self-discrepancies). Participants were exposed to priming based on 3 different conditions (i.e., relevant but not discrepant attributes, relevant and discrepant attributes and a yoked condition). Results showed that only those participants exposed to a discrepancy-relevant priming condition showed a change in their levels of certain emotions (i.e., dejected or agitation) depending on the type of predominantly primed discrepancy and the type of relevant self-discrepancy of each participant. Therefore, according to this type of former evidence, discrepancies between the actual-self and other domains could serve as indicators of predominantly active latent cognitive schemas, which, when interacting with contextual stressors, could facilitate an increase in the salience of such mismatch, thus generating the characteristic emotional responses to each type of self-discrepancy (27). Consequently, the way in which the individual defines him/herself and, above all, the degree of discrepancy with their other self-domains (i.e., ideal or ought) seem to be a variable of relevance to study the emergence of emotional symptomatology, and their potentially intervening mechanisms (i.e., resulting negative interpretation biases).

**Figure 1:**
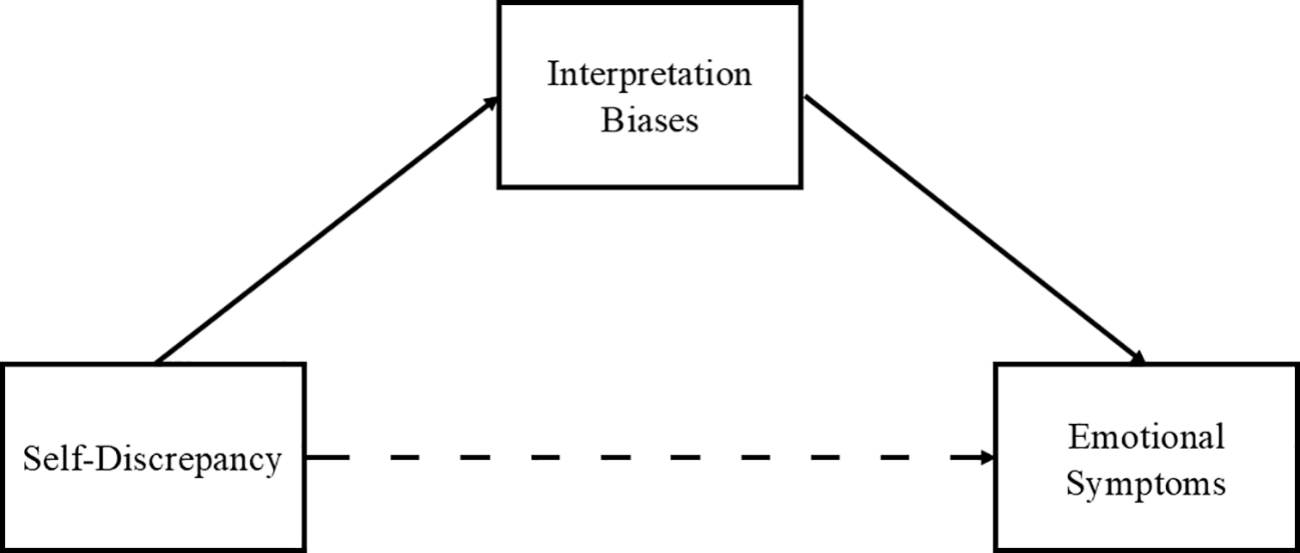
Graphical depiction of the model tested.

Consequently, in this study, we aimed to test the explanatory mechanisms of emotional symptoms from traditional cognitive models (e.g., existence of a latent cognitive scheme that would produce biases in the processing of information when interacting with a stressor) through the evaluation of these schemes in relation to the existence of self-discrepancies. The integration of these frameworks could help to improve the understanding of causal factors for the occurrence of emotional symptoms. Yet, the relationships between self-discrepancies and interpretation biases and their specific paths of influence in changes in emotional symptoms have not been previously tested yet. Thus, we tested mediational models through which self-discrepancies would act as latent self-referential cognitive schemas, which would facilitate higher levels of negative self-referential interpretation biases and, consequently, explaining the presence and change of emotional symptoms. Specifically, we analyzed the relationships between individual differences in self-discrepancies (i.e., actual-ideal, ought-ideal), negative interpretation biases, and emotional symptoms (i.e., depression, anxiety), considering the mediating role of negative interpretation biases in the relationship between self-discrepancies and emotional symptoms both cross-sectionally and longitudinally. To do this, undergraduate participants were asked to perform two tasks, one assessing self-discrepancies [i.e., actual-ideal self-discrepancies and actual-ideal self-discrepancies; (28) and another one assessing negative interpretation biases (29)]. In order for participants to respond based on how they perceived themselves, both tasks used clearly self-referential stimuli. Similarly, symptoms of depression and anxiety were assessed after completing the tasks and approximately one-two month later, right before starting undergraduates’ exam period, which previous research has identified as a naturalistic major stressor for the undergraduate population under study (30). This methodology allowed us to test individual differences in the change in symptoms when confronting a specific stress situation (**Figure 1**). Overall, it was hypothesized that higher levels of self-discrepancies would have a significant and positive direct relationship with higher negative interpretation biases, such that the greater the magnitude of the self-discrepancy, the higher the level of the bias. Furthermore, it was hypothesized that higher negative interpretation biases would have a mediating role in the relationship between higher self-discrepancies and higher emotional symptom levels.

## 2 Materials and methods

### 2.1 Research type and design

This study employs a quantitative, observational, non-experimental approach with a mediational and longitudinal design. This design was used to examine the role of negative interpretation biases as a mediator in the relationships between self-discrepancies (actual-ideal and actual-ought) and emotional symptoms (depression and anxiety). The cross-sectional analysis was conducted to test the relationships between variables at a single time point, while the longitudinal analysis tested these relationships when assessing changes in emotional symptoms over time, specifically in response to a natural stressor (exam period).

### 2.2 Ethical aspects

The study was conducted in accordance with the ethical guidelines of the Declaration of Helsinki. Approval was obtained from the university’s ethics committee (CE_20240111_20_SAL). Participants were informed about the objectives of the research and provided written informed consent before participating. They were assured of the confidentiality and anonymity of their data, as well as their right to withdraw from the study at any time without any consequences. Additionally, all participants received course credits as compensation for their involvement in the study.

### 2.3 Population, sample and sampling

The sample was composed by 73 (61 females, 12 males) university students from Complutense University of Madrid with a mean age of 21.45 years (SD = 3.57). Of the total sample, 72 participants (60 females; mean age = 21.47; SD age = 3.59) completed a follow-up in which their levels of emotional symptoms were assessed again just before the beginning of the exam period, an event that has been identified as a relevant stressor for university students (30). The inclusion criteria for the study were as follows: (1) undergraduate students enrolled at Complutense University of Madrid, (2) native Spanish speakers, and (3) individuals with normal or corrected-to-normal vision. Recruitment was conducted by advertising the study in various classes, offering university credits as compensation.

### 2.4 Instruments

#### 2.4.1 Questionnaires

##### 2.4.1.1 Depressive symptoms

The Patient Health Questionnaire (PHQ-9) (31, 32) is a nine-item questionnaire that evaluates depressive symptoms’ severity during the previous two weeks in a four-point scale (from 0 to 27). Higher values indicate higher levels of depressive symptoms. In this study, the PHQ-9 showed a high internal consistency at both the baseline (α = .84) and the follow up (α = .88).

##### 2.4.1.2 Anxiety symptoms

The Generalized Anxiety Disorder Scale (GAD-7) (33, 34) is a seven-item questionnaire that measure the severity of anxiety symptoms in the previous two weeks in a four-point scale (from 0 to 21). Higher values indicate higher severity of anxiety symptoms. In this study, the GAD-7 showed a high internal consistency at both the baseline (α = .89) and the follow-up (α = .89).

#### 2.4.2 Experimental tasks

##### 2.4.2.1 Self-discrepancies

In order to evaluate the different types of self-discrepancies based on Higgins’ model (23–25), the SCQ-CC [Self-Concept Questionnaire-Conventional Construct: (28)] was used. This task allows evaluating the different self-domains (i.e., actual, ideal and ought) to calculate the differences between them, thus generating self-discrepancy indices [i.e., actual-ideal self-discrepancy and actual-ought self-discrepancy; see (23–25, 27)]. To do this, participants had to rate 28 self-referent adjectives (e.g., cheerful, worrying, organized, warm), on a scale from 1 to 7, to what extent those attributes described them for each of the different types of self-domains defined by Higgins’ theory (i.e., actual, ideal and ought), forming a total of 84 items. For this study, the task thus focused on assessing the participant’s own perception of him/herself, not including other standpoints such as the participant’s idea of how other relevant people think about him/her (i.e., standpoint ‘others’). Therefore, the discrepancy indices calculated were referred to the individual’s perspective on himself (actual own-ideal own self-discrepancy and actual own-ought own self-discrepancy) (23–25).

Self-discrepancies were calculated following the same method as in previous studies [e.g., (28)]. In this case, actual-ideal self-discrepancy was calculated averaging the absolute difference between the scores of the self-reports from actual and ideal self. The actual-ought discrepancy was calculated in the same way, but only referring to the absolute differences between the scores of the self-reports from actual and ought self. Thus, two self-discrepancy indices were computed for each participant, actual-ideal and actual-ought, that ranged from 0 (no discrepancy) to 6 (maximum discrepancy). In this task, the self-discrepancies index showed good internal consistencies, for both actual-ideal (α = .76) and actual-ought (α = .80).

##### 2.4.2.2 Interpretation bias

**Figure 2:**
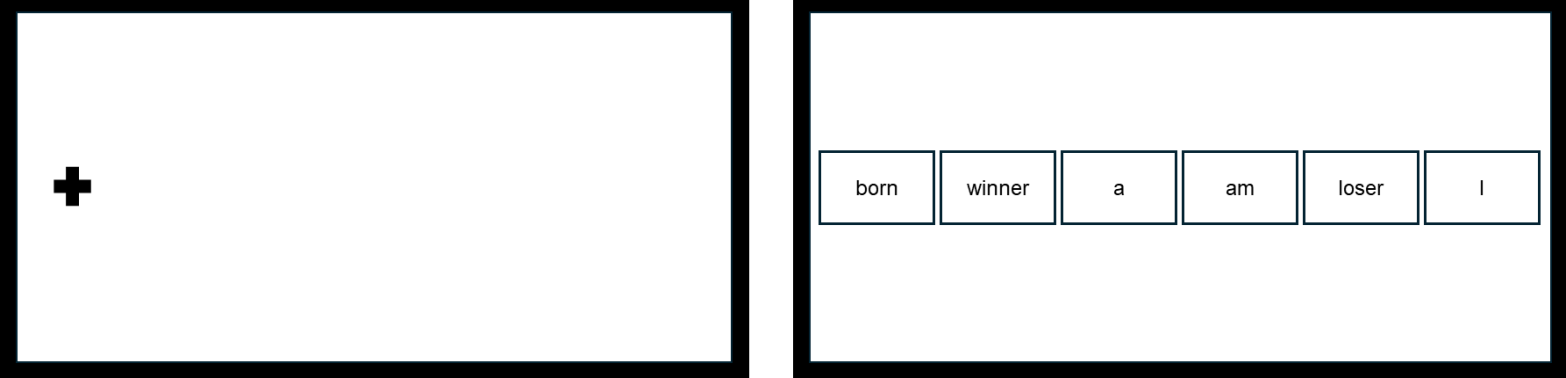
Reading phase from SST.

**Figure 3:**
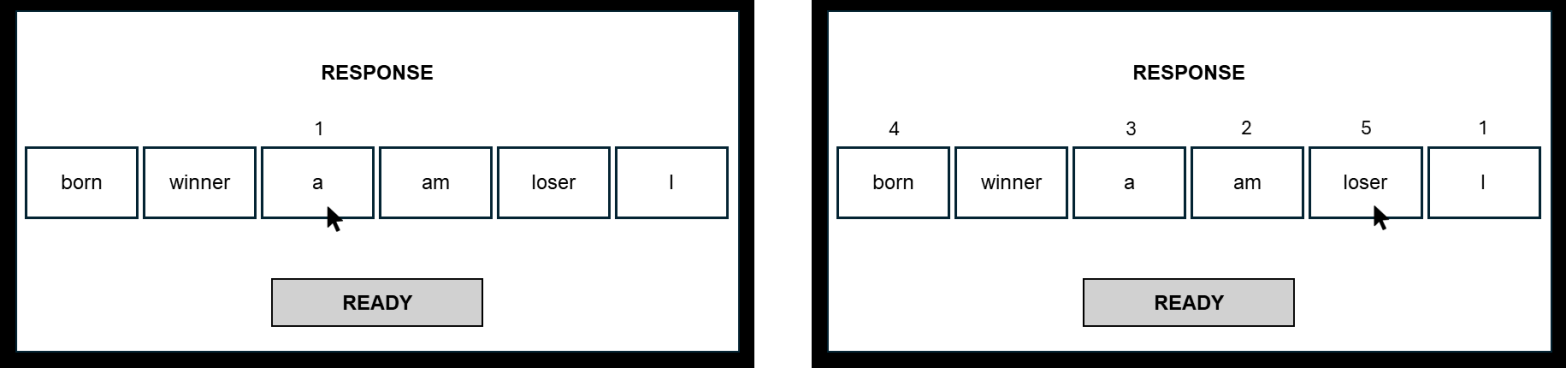
Response phase from SST.

To assess the degree of negative interpretation biases, a computerized version of the Scramble Sentence Task (SST) (29) was used. In this task, participants must form grammatically correct sentences with 5 out of the 6 words that appear scrambled on the screen, and that can be solved into positive or negative unscrambled sentences, thus assessing the participants’ tendency to solve ambiguous material in specific emotional ways. This task has demonstrated its validity to evaluate negative interpretation bias in other studies with similar methodology (21, 35–37), and shown capacity to differentiate the degree of biases at different stages of emotional problems (i.e., dysphoric, clinically depressed, formerly depressed) (21). The task for this study comprised 30 items. All trials started with a fixation cross on the left side of the screen during 1500 ms, which the participants were asked to look at. Next, the reading phase started (**Figure 2**), where participants had to read from left-to-right the 6 words that appeared for 8000 ms, in order to mentally unscramble the material to form a grammatically correct sentence using only 5 out of those words. After this time, the response phase began (**Figure 3**) and the participants, using the mouse, had to click over the different boxes where the words appeared in order to form the previously unscrambled sentence. To do this, they had a limited time of 9000 ms. After that time limit, if a full response had not been indicated, the task moved to the next trial. Previous to complete the actual task, participants had to complete 5 practice items based on unscrambling neutral sentences (e.g., ‘I really like eating grapes/bananas’), to get familiar with the procedure, before completing the 30 emotional items of the main task.

The emotional sentences of the task (e.g., born winner a am loser I) could be solved into a positive (e.g., I am a born winner) or a negative (e.g., I am a born loser) solution. Thus, the number of negatively unscrambled sentences was divided by the total number of unscrambled sentences to index the tendency of participants to interpret ambiguity in a negative manner. To avoid a bias due to the position of the emotional words, it was controlled that negative and positive words appeared the same proportion of times in left- and right-positions of the screen (second and fifth boxes) across the trials. The negative interpretation bias index ranged from 0 to 1, with higher values indicating a higher negative interpretation bias. To obtain an accurate index, only those participants who solved at least 20 sentences out of the 30 sentences correctly were included. Using this criterion, no participants had to be eliminated since all of them completed at least 20 trials correctly.

### 2.5 Procedure

Upon arriving at the laboratory, participants were informed about the purpose of the study and were asked to sign the informed consent form if they agreed to participate. They were then instructed to start completing the paper-and-pencil version of the SQC-CC (28), followed by the SST (29) via e-Prime 3.0, and finally to complete the online questionnaires assessing depression and anxiety via Qualtrics. The experimental session lasted less than 60 minutes on average. Approximately one-two months later, just before the exam period, participants were recontacted and asked to complete the same set of questionnaires as during the experimental session to obtain the longitudinal measures of symptoms’ change.

### 2.6 Data-analysis plan

To assess the relationships between self-discrepancies, interpretation biases, and emotional symptoms, a series of steps were taken based on the established hypotheses. First, correlations between the study’s relevant variables (self-discrepancies, interpretation biases, and emotional symptoms) were calculated using SPSS version 27.0 to evaluate the initial relationships between them. Next, mediation models were constructed with the key variables at T1, where self-discrepancies served as the independent variables, negative interpretation bias as the mediator, and emotional symptoms (depression and anxiety) as the dependent variables. Standardized indices were calculated to facilitate the interpretation of the different effects tested in the models (i.e., direct and indirect effects). Further, the same models were tested longitudinally (i.e., considering changes in emotional symptoms from T1 to T2, at the confrontation of the stressor). To do this, standardized residuals of emotional symptoms at T1 predicting themselves at T2 were computed, as indices of change in depression and anxiety at the face of stress. These indices of change were then entered as the dependent variables in the longitudinal models, allowing to test how self-discrepancies at T1 predicted emotional symptoms’ changes, directly and indirectly through the mediation of negative interpretation biases. Standardized residuals are a widely used method for assessing change beyond simple differences between time points (38, 39). Specifically, this parameter is obtained by calculating the residuals resulting from performing a linear regression where the symptoms (whether depressive or anxious) at T1 predict the symptoms (whether depressive or anxious) at T2. Thus, the obtained standardized residual values represent the proportion of variance in symptoms’ change not explained by the previous levels of symptomatology (i.e., T1 scores)^1^. All mediation models were computed using JASP version 18.2.

## 3 Results

Bivariate correlations between the variables of the study were first computed. Results indicated that both types of self-discrepancies were related with higher levels of depressive ( $r_{act-ide\;SD}\;=\;.51,\;p\;<\;.001$ ; $r_{act-oug\;SD}\;=\;.53,\;p\;<\;.001$ ), anxiety symptoms ( $r_{act-ide\;SD}\;=\;.56,\;p\;<\;.001$ ; $r_{act-oug\;SD}\;=\;.52,\;p\;<\;.001$ ) and with higher levels of negative interpretation biases ( $r_{act-ide\;SD}\;=\;.56,\;p\;<\;.001$ ; $r_{act-oug\;SD}\;=\;.54,\;p\;<\;.001$ ) at T1. In the same way, higher levels of negative interpretation biases were related with higher levels of depressive (*r* = .65, *p*<.001) and anxiety (*r* = .56, *p*<.001) symptoms. Both types of self-discrepancies were highly related (*r* = .74, *p*<.001). By last, no discrepancy indices nor negative interpretation bias were related to symptom change indices from T1 to T2 (all r’s<.14 & > -.07). In **Supplementary Table 1** appears the mean and standard deviation and in **Supplementary Table 2** the specific correlation indices of variables implicated in the study.

### 3.1 Cross-sectional mediation models

#### 3.1.1 Cross-sectional models with actual-ideal self-discrepancies as predictor

After initially verifying a relationship between the study variables, it was decided to carry out mediational models with each of the self-discrepancy variables as predictors, negative interpretation bias as the mediator and emotional symptoms’ indices as outcome measures. Following (17, 35, 37, 40), mediational effects were tested through bootstrapping procedures. In this case, 95% confidence intervals with 5000 bias-corrected bootstrap samples were estimated, thus an indirect effect was significant if the confidence interval did not include 0.

**Figure 4:**
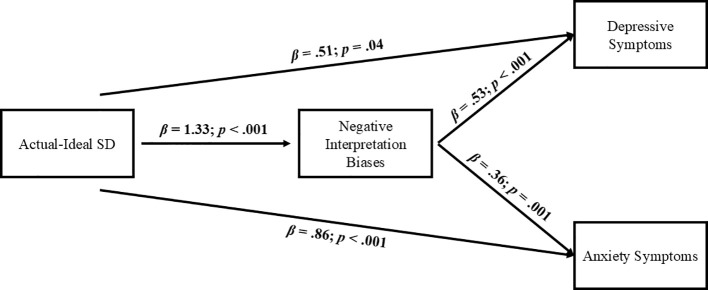
Cross-sectional mediational model with actual-ideal SD as first predictor. SD, Self-Discrepancy.

As for the models considering the actual-ideal self-discrepancy as the predictor, its direct effect was positive and significant for both types of symptoms: depressive (β:.51, *SE*:.25, *z* = 2.05, *p* = .04) and anxious symptom levels (β:.86, *SE*:.26, *z* = 3.3, *p*<.001). Likewise, a higher actual-ideal self-discrepancy was also significantly related to higher levels of negative interpretation bias (β:1.33, *SE*:.23, *z* = 5.72, *p*<.001). Finally, a higher negative interpretation bias was related to higher levels of both depressive (β:.53, *SE*:.1, *z* = 5.003, *p*<.001) and anxiety symptoms (β:.36, *SE*:.11, *z* = 3.29, *p* = .001). **Figure 4** shows the different direct relationships between the study variables.

Regarding the indirect effects, the analyses supported a significant indirect effect of negative interpretation bias in the relationship between the actual-ideal self-discrepancy and depression (*β*:.7, *SE*:.19, *z* = 3.77, *p*<.001, CI:.37, 1.16) and anxiety symptom levels (*β*:.48, *SE*:.17, *z* = 2.85, *p* = .004, CI:.18,.95), thus supporting that that actual-ideal self-discrepancies were related to both forms of symptomatology indirectly through their relation with a higher activation of negative interpretation biases.

#### 3.1.2 Cross-sectional models with actual-ought self-discrepancies as predictor

**Figure 5:**
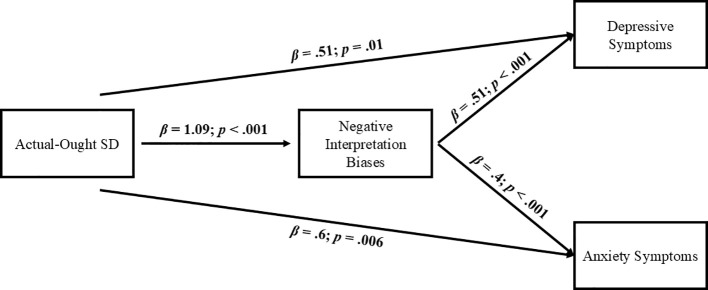
Cross-sectional mediational model with actual-ought SD as first predictor. SD, Self-Discrepancy.

As for the actual-ought self-discrepancies, the results showed a similar pattern. In this case, actual-ought self-discrepancies were positive and directly related to both depressive (*β*:.51, *SE*:.21, *z* = 2.5, *p* = .01) an anxiety symptom levels (*β*:.6, *SE*:.22, *z* = 2.72, *p* = .006), as well as with negative interpretation biases (*β*: 1.09, *SE*:.2, *z* = 5.44, p<.001). In the same way, negative interpretation biases were highly associated with both depressive (*β*:.51, *SE*:.1, *z* = 4.98, *p*<.001) and anxiety (*β*:.4, *SE*:.11, *z* = 3.63, *p*<.001) symptoms (**Figure 5**).

Regarding the indirect effects of these models, the results supported significant indirect effects of negative interpretation bias in the relationship between actual-ought self-discrepancies and both forms of emotional symptoms, depression (*β*:.55, *SE*:.15, *z* = 3.67, *p*<.001, *CI*:.32,.87) and anxiety (*β*:.43, *SE*:.14, *z* = 3.02, *p* = .003, *CI*:.17,.82). Therefore, although there was a direct effect of the level of actual-ought self-discrepancies on both forms of symptomatology, part of this effect occurred indirectly through their influence on a higher activation of negative interpretation biases.

### 3.2 Longitudinal mediation models

#### 3.2.1 Longitudinal models with actual-ideal self-discrepancies as predictor

**Figure 6:**
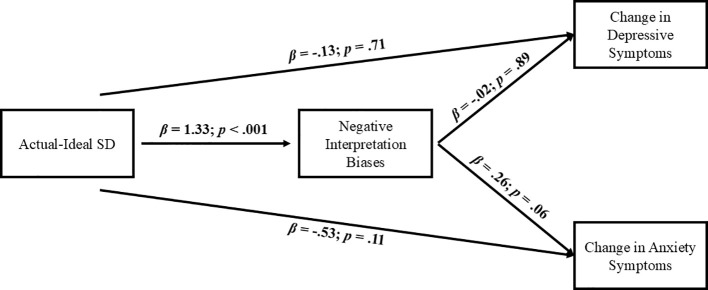
Longitudinal mediational model with actual-ideal SD as first predictor. SD, Self-Discrepancy.

Focusing on the longitudinal model (i.e., testing the mediation role of negative interpretation bias in the relation between actual-ideal self-discrepancies and the change in emotional symptoms from T1 to T2, indexed through standardized residuals), the results showed differences with respect to the ones from the cross-sectional models. In this case, results showed that higher actual-ideal self-discrepancies at T1 were not directly related to changes in emotional symptoms for both depression (*β*: -.13, *SE*:.34, *z* = -.38, *p* = .71; and anxiety symptom (*β*: -.53, *SE*:.33, *z* = -1.6, *p* = .11), while their association with higher levels of negative interpretation bias remained significant (*β*: 1.33, *SE*:.23, *z* = 5.72, *p*<.001). Negative interpretation bias did not predict changes in depressive symptoms (*β*: -.02, *SE*:.14, *z* = -.13, *p* = .89), although there was a trend to predict increases in anxiety symptoms (*β*:.26, *SE*:.14, *z* = 1.91, *p* = .06) (**Figure 6**).

Regarding the indirect effects, the results supported a mediational effect of higher actual-ideal self-discrepancies predicting increases in anxiety symptoms through their relationship with a higher activation of negative interpretation biases (*β*:.35, *SE*:.19, *z* = 1.81, *p* = .07, *CI*:.03,.71). In contrast, this mediational effect was not supported for the model considering changes in depression symptom levels (*β*: -.02, *SE*:.19, *z* = -.13, *p* = .89, *CI*: -.37,.32).

#### 3.2.2 Longitudinal models with actual-ought self-discrepancies predictor

**Figure 7:**
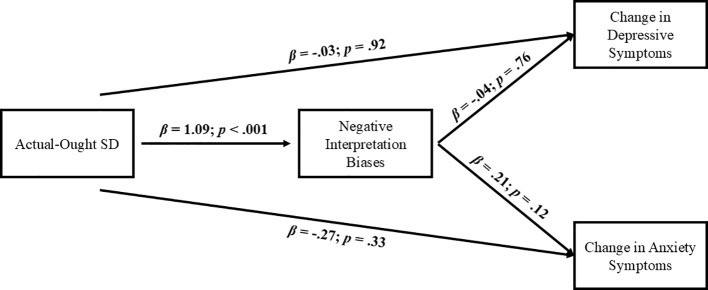
Longitudinal mediational model with actual-ought SD as first predictor. SD, Self-Discrepancy.

Finally, longitudinal models were conducted considering actual-ought self-discrepancies as the predictor (i.e., testing the mediation role of negative interpretation bias in the relation between actual-ought self-discrepancies and the change in emotional symptoms measured through standardized residuals). The direct effect of actual-ought self-discrepancies on the change in emotional symptomatology, either depressive (*β*: -.03, *SE*:.28, *z* = -.1, *p* = .92) or anxious symptom levels (*β*: -.27, *SE*:.28, *z* = -.98, *p* = .33) were both not significant. The association between actual-ought self-discrepancies and negative interpretation bias remained significant (*β*: 1.09, *SE*:.2, *z* = 5.44, *p*<.001). Negative interpretation biases did not predict the change in emotional symptoms, for both depression (*β*: -.04, *SE*:.14, *z* = -.3, *p* = .76) and anxiety (*β*:.21, *SE*:.14, *z* = 1.55, *p* = .12) (**Figure 7**).

As for indirect effects, they were both non-significant, indicating that actual-ought self-discrepancies did neither predict changes in emotional symptoms through their relationship with negative interpretation bias (*β*: -.04, *SE*:.15, *z* = -.3, *p* = .76, *CI*: -.27,.22; *β*:.23, *SE*:.15, *z* = 1.49, *p* = .14, *CI*: -.06,.59, for depression and anxiety, respectively).

## 4 Discussion

The way we define ourselves, especially the differences between how we actually perceive ourselves in comparison to other self-domains (e.g., ideal or ought), seems to be a key variable in understanding the experience of multiple emotional responses. Self-discrepancies can be understood as latent self-referential cognitive schemas, increasing certain cognitive biases in emotional information processing. In turn, a higher degree of these biases can facilitate more maladaptive emotional dynamics (23, 25–27). However, no previous studies have integrated the study of all these processes together. In consequence, in this study we aimed to analyze the relationships between these variables and test the capacity of self-discrepancies to activate specific information processing biases (i.e., negative interpretation biases), and whether this would result in higher levels of emotional symptoms. Overall, the results support the mediating role of negative interpretation bias in the relationship between higher self-discrepancies and higher emotional symptom levels. Greater self-discrepancies were related to a pattern of more biased negative information processing, which in turn was related to higher levels of emotional symptoms. This mediation was fully supported for both types of self-discrepancies in cross-sectional models and was partially supported in longitudinal models. The indirect effect of negative interpretation bias was specifically supported only for the role of actual-ideal self-discrepancies in predicting changes in anxiety symptoms. These results are discussed below.

### 4.1 Support of Higgins’ model

First, in this study we aimed to analyze the support of predictions from Higgins’ framework on the role of different self-discrepancies on different forms of emotional symptomatology (23–25). The results indicated that, contrary to the specificity proposed by this model (i.e., higher actual-ideal self-discrepancy being related to higher depressive symptoms and higher actual-ought self-discrepancy being related to higher anxiety symptoms), both self-discrepancy indices were related to higher levels of both types of emotional symptoms. These relationships were supported by correlational and cross-sectional mediation analyses. This inconsistency with the model’s prediction of specificity has been observed in other studies, where various combinations of relationships between self-discrepancies and emotion symptoms have been found. For example, some studies support the association of actual-ideal discrepancy with both types of symptoms (41, 42), while other studies support the associations of actual-ought self-discrepancy with only depressive symptoms (43) or both emotional symptoms (44). Further, other previous studies have not found differences in actual-ideal and actual-ought self-discrepancies between clinically depressed and anxious participants (45). This contrast with the results of other studies that fully support Higgins’ model assumptions on specificity (23–25, 46, 47). These apparent inconsistency between results can be influenced by the type of task used in each study. Some studies have relied in idiographic tasks (i.e., assessing self-discrepancies through the election of unique personal traits/adjectives that define their understanding of themselves) whereas others make use of nomothetic tasks (i.e., assessing self-discrepancies based on a preestablish list of traits/adjectives). Some authors support the former (46), while others claim a high correlation between the indices, arguing the use of both types of tasks (28, 48). Consequently, the results might suggest the existence of a transdiagnostic variable rather than multiple subindexes associated with specific symptomatic patterns (49).

### 4.2 Support of the relation between self-discrepancy indices and interpretation biases

As part of the central hypotheses of the study, the effect of self-discrepancies on promoting specific negative information processing biases were tested. The results support this assumption, showing a high degree of association between both types of self-discrepancies and negative interpretation biases across all types of analysis. None of the specific discrepancy indices exhibited a greater or lesser relationship with negative interpretation bias levels. In future studies, different types of negative interpretation bias (i.e., regarding solving ambiguity for specifically relevant depression and anxiety topics) could provide additional information regarding the specificity on relationships between self-discrepancies and negative interpretation biases. This relationship between self-referential discrepancies and other forms of negative interpretation biases has been observed in studies on eating disorders, where patients exhibited a negative bias in their body interpretation. In fact, this interpretation bias was correlated with body dissatisfaction, a key aspect in defining identity (50). Thus, these results suggest the potential importance of self-referential discrepancies (23–25) to explain the emergence and maintenance of emotional symptoms, as they may reflect specific activation of negative cognitive schemas (22). Following that logic, our results (i.e., through correlational and cross-sectional mediational analysis) can be seen as a further support for predictions from traditional cognitive models, which state that cognitive schemas produce changes in information processing patterns through cognitive biases, leading to characteristic emotional symptoms (22).

### 4.3 Support of the mediational role of interpretation biases in the relationship between self-discrepancies and emotional symptoms

Lastly, the mediating role of interpretation bias in the relationship between self-discrepancies and emotional symptoms was tested. The results support the predictive value of higher negative interpretation bias on higher levels of both types of emotional symptoms cross-sectionally, yet only for changes in anxiety in the longitudinal mediational models. Cross-sectional results are supported by other studies that have found direct associations between negative interpretation bias and both forms of emotional symptoms [e.g., (15, 17, 19, 20)]. However, the longitudinal value of cognitive biases to predict both forms of symptoms has not been deeply explored in previous literature, with some studies supporting its predictive value for both forms of symptoms (20, 51, 52), while others do not reach that assumption so clearly (53). These results could be due to several differences among studies, such as the type of sample investigated (i.e., clinical vs. subclinical), the lack of consideration of emotion regulation strategies or contextual factors influencing data collection. Regarding this, multiple models propose that the effect of biased information processing to predict changes in symptoms would depend on how biases influence subsequent emotional regulation processes, rather than directly influencing on the symptoms (e.g., 12). Another reason for the only prediction on anxiety change could be due to the contextual component of the study, as the longitudinal data collection was designed to detect symptom changes in response to an upcoming stressful event (the exam period). For this reason, it is possible that the time at which the data were collected may had particularly exacerbated changes in anxiety when facing an imminent stressful situation (e.g., exam period) as a common mechanism when trying to face an event that is interpreted as challenging. Following this logic, a better detection of individual differences in changes in depressive symptoms as a result of this particular stressor, would require to further assess emotional symptoms at the end of the event (i.e., after having completed the exam period and getting the final grades). This could at least partly explain a higher room to detect changes in anxiety and the mechanisms implicated in predicting that change in the current study, contrasting with the null results found for the longitudinal mediational models considering depression change as an outcome.

Overall, whereas there are previous studies evaluating the role of discrepancies as cognitive schemas on emotional symptoms, none to date have integrated the study of their influence on negative information processing and the various mediating effects of the latter on the relationships between self-discrepancies and emotional symptoms. Moreover, conducting not only cross-sectional but also longitudinal models allowed us to test the predictive value of discrepancies and biases in symptom change. The study thus holds significant clinical value by providing initial insights into how identity-related variables can influence the processing of ambiguous information, thereby potentially triggering emotional symptoms in the face of a stressor. From a clinical perspective, there are multiple implications associated with the study. Firstly, although longitudinal models did not show clear results, different mechanisms through which intervention could be designed to reduce emotional symptoms were observed. Both self- discrepancies (associated with a self-referential component) and negative interpretation biases seem to be linked to each other, so changes in one could cause changes in the other. Therefore, the management or resolution of self-discrepancies (e.g., cognitive behavioral therapy) could facilitate the reduction of negative biases. Similarly, interventions designed to modify these cognitive biases (54) has shown how such reduction can relate to improvements in emotional symptoms which could in turn influence the future activation of negative cognitive schemes.

## 5 Limitations

Despite the relevance of these findings, several limitations must be considered. Firstly, the sample consisted predominantly of undergraduate students, mostly female. This limits the representativeness of the findings and reduces the generalizability to broader social groups (e.g., individuals of different ages, occupations, and stress levels). In consequence, results may reflect the experiences of a specific social group rather than those ones of the general population, due to both the sample size and its characteristics. Secondly, the study focuses on an educational context, particularly the exam period as a natural stressor. This situation may be too specific to students and may not generalize to other social groups or life contexts. This limits the extrapolation of results to other types of stressors (e.g., workplace or family-related stress), which could activate different cognitive schemas and yield different interpretation biases. These limitations warrant further replication in broader samples and considering different forms of stress context in future research. Thirdly, other limitations arise from the types of statistical analyses used in the study. The accuracy of mediational models is sensitive to sample size, which in this study may lead to findings that are specific to the sample rather than reflective of a broader population. With a small sample size, the power to detect subtle effects might be reduced, and the chance of sample-specific results increased. Although bootstrapping was employed to enhance the robustness of results’ estimation, a larger sample would provide a more reliable basis for testing specific mediational relationships among these factors in future studies. Finally, the timing of longitudinal data collection may have influenced results, as the assessment of emotional symptom changes occurred just before the stressor onset. This timing might have contributed to the detection of changes primarily in anxiety rather than depression, potentially limiting the assessment of the predictive roles of self-discrepancies and negative interpretation biases in depression specifically.

As such, our results should be interpreted with caution. Future studies should increase sample size and diversify the sample to include different social characteristics (e.g., a more balanced gender ratio and a broader age range), providing a more robust pattern of results. Additionally, future research should consider testing the relationships between self-discrepancies and negative interpretation biases in clinical populations. Such studies could yield valuable insights into how cognitive schemas and biases may contribute to the maintenance or recurrence of emotional symptoms following specific stressors. Further, adding a follow-up assessment of emotional symptoms at the stressor’s conclusion (e.g., after the exam period and receipt of final grades) could capture a wider range of symptom variability, allowing distinctions between individuals who recover from the stressor and those whose symptoms persist or intensify. This expanded time frame would provide a more precise temporal context for analyzing how self-referential mechanisms predict emotional symptom dynamics over time, offering a clearer perspective on symptom change patterns.

## 6 Conclusions

In conclusion, the results of this study show the importance of self-discrepancies as self-referential mechanisms that are relevant in explaining emotional symptoms. Results support the idea that self-discrepancies could be conceptualized as facets of the activation of latent cognitive schemas that would in turn facilitate the activation of negative biases during the processing emotional information, lastly influencing the appearance and/or maintenance of different emotional symptoms.

## Data Availability

The raw data supporting the conclusions of this article will be made available by the authors, without undue reservation.

## Footnotes

1 With the aim to test if the exam period produced the expected effect in participants in depression and anxiety symptoms between the baseline period (T1) and the follow-up period (T2 = exam period), a series of repeated measures ANOVAs was carried out. Results showed no significant changes in depressive (F = 3.54, p = .06, η_p_^2^ = .05) or anxious (F = 1.19, p = .28, η_p_^2^ = .02) symptoms.

## References

[1] Gutiérrez-Rojas L Porras-Segovia A Dunne H Andrade-González N Cervilla JA. Prevalence and correlates of major depressive disorder: a systematic review. Braz J Psychiatry. (2020) 42(6):657–72. doi: 10.1590/1516-4446-2020-0650 PMC7678895 32756809

[2] Javaid SF Hashim IJ Hashim MJ Stip E Samad MA Ahbabi AAl. Epidemiology of anxiety disorders: global burden and sociodemographic associations. Middle East Curr Psychiatry. (2023) 30(1):44. doi: 10.1186/s43045-023-00315-3

[3] Moreno-Agostino D Wu Y.-T Daskalopoulou C Hasan MT Huisman M Prina M. Global trends in the prevalence and incidence of depression:a systematic review and meta-analysis. J Affect Disord. (2021) 281:235–43. doi: 10.1016/j.jad.2020.12.035 33338841

[4] ten Doesschate MC Koeter MWJ Bockting CLH Schene AH. Health related quality of life in recurrent depression: A comparison with a general population sample. J Affect Disord. (2010) 120(1-3):126–32. doi: 10.1016/j.jad.2009.04.026 19446342

[5] Zhang J Li Z. The association between depression and suicide when hopelessness is controlled for. Compr Psychiatry. (2013) 54:790–6. doi: 10.1016/j.comppsych.2013.03.004 PMC3745521 23602028

[6] Santini ZI Koyanagi A Tyrovolas S Mason C Haro JM. The association between social relationships and depression: A systematic review. J Affect Disord. (2015) 175:53–65. doi: 10.1016/j.jad.2014.12.049 25594512

[7] König H König H-H Konnopka A. The excess costs of depression: a systematic review and meta-analysis. Epidemiol Psychiatr Sci. (2020) 29:e30. doi: 10.1017/S2045796019000180 PMC8061284 30947759

[8] Konnopka A König H. Economic burden of anxiety disorders: A systematic review and meta-analysis. PharmacoEconomics. (2020) 38:25–37. doi: 10.1007/s40273-019-00849-7 31646432

[9] Vieta E Alonso J Pérez-Sola V Roca M Hernando T Sicras-Mainar A. Epidemiology and costs of depressive disorder in Spain: the EPICO study. Eur Neuropsychopharmacol. (2021) 50:93–103. doi: 10.1016/j.euroneuro.2021.04.022 34058711

[10] Gross JJ Thompson RA. Emotion regulation : conceptual foudantions. Handb Emotion Regul. (2007), 3–24.

[11] De Raedt R Koster EHW. Understanding vulnerability for depression from a cognitive neuroscience perspective: A reappraisal of attentional factors and a new conceptual framework. Cogn Affect Behav Neurosci. (2010) 10:50–70. doi: 10.3758/CABN.10.1.50 20233955

[12] Joormann J Vanderlind WM. Emotion regulation in depression: The role of biased cognition and reduced cognitive control. Clin psychol Sci. (2014) 2:402–21. doi: 10.1177/2167702614536163

[13] Mehu M Scherer KR. The appraisal bias model of cognitive vulnerability to depression. Emotion Rev. (2015) 7:272–9. doi: 10.1177/1754073915575406

[14] Gross JJ Uusberg H. Mental illness and well-being: an affect regulation perspective. World Psychiatry. (2019) 18:130–9. doi: 10.1002/wps.20618 PMC6502417 31059626

[15] Everaert J Podina IR Koster EHW. A comprehensive meta-analysis of interpretation biases in depression. Clin Psychol Rev. (2017) 58(4):33–48. doi: 10.1016/j.cpr.2017.09.005 28974339

[16] Chen J Short M Kemps E. Interpretation bias in social anxiety: A systematic review and meta-analysis. J Affect Disord. (2020) 276:1119–30. doi: 10.1016/j.jad.2020.07.121 32777650

[17] Everaert J Grahek I Duyck W Buelens J Van den Bergh N Koster EHW. Mapping the interplay among cognitive biases, emotion regulation, and depressive symptoms. Cogn Emotion. (2017) 31:726–35. doi: 10.1080/02699931.2016.1144561 26878897

[18] Romano M Moscovitch DA Saini P Huppert JD. The effects of positive interpretation bias on cognitive reappraisal and social performance: Implications for social anxiety disorder. Behav Res Ther. (2020) 131:103651. doi: 10.1016/j.brat.2020.103651 32504886

[19] Blanco I Boemo T Sanchez-Lopez A. An online assessment to evaluate the role of cognitive biases and emotion regulation strategies for mental health during the COVID-19 lockdown of 2020: structural equation modeling study. JMIR Ment Health. (2021) 8:e30961. doi: 10.2196/30961 34517337 PMC8565804

[20] Wisco BE Harp DR. Rumination as a mechanism of the association between interpretation bias and depression symptoms: A longitudinal investigation. J Exp Psychopathol. (2021) 12:204380872110152. doi: 10.1177/20438087211015233

[21] Martin-Romero N Sanchez-Lopez A. Negative interpretation bias as a clinical marker and a scar of depression: New insights from a large-scale study of the scrambled sentence task in formerly, subclinically and clinically depressed individuals. Behav Res Ther. (2023) 163:104276. doi: 10.1016/j.brat.2023.104276 36821874

[22] Beck AT Haigh EAP. Advances in cognitive theory and therapy: the generic cognitive model. Annu Rev Clin Psychol. (2014) 10:1–24. doi: 10.1146/annurev-clinpsy-032813-153734 24387236

[23] Higgins ET Klein R Strauman T. Self-concept discrepancy theory: A psychological model for distinguishing among different aspects of depression and anxiety. Soc Cogn. (1985) 3:51–76. doi: 10.1521/soco.1985.3.1.51

[24] Higgins ET Bond RN Klein R Strauman T. Self-discrepancies and emotional vulnerability: How magnitude, accessibility, and type of discrepancy influence affect. J Pers Soc Psychol. (1986) 51(1):5–15. doi: 10.1037/0022-3514.51.1.5 3735070

[25] Higgins ET. Self-discrepancy: A theory relating self and affect. psychol Rev. (1987) 94:319–40. doi: 10.1037/0033-295X.94.3.319 3615707

[26] Klenk MM Strauman TJ Higgins ET. Regulatory focus and anxiety: A self-regulatory model of GAD-depression comorbidity. Pers Individ Dif. (2011) 50:935–43. doi: 10.1016/j.paid.2010.12.003 PMC3079259 21516196

[27] Strauman TJ Higgins ET. Automatic activation of self-discrepancies and emotional syndromes: when cognitive structures influence affect. J Pers Soc Psychol. (1987) 53:1004–14. doi: 10.1037/0022-3514.53.6.1004 3694448

[28] Watson N Bryan BC Thrash TM. Self-discrepancy: Comparisons of the psychometric properties of three instruments. psychol Assess. (2010) 22:878–92. doi: 10.1037/a0020644 21133548

[29] Wenzlaff RM Bates DE. Unmasking a cognitive vulnerability to depression:How lapses in mental control reveal depressive thinking. J Pers Soc Psychol. (1998) 75:1559–71. doi: 10.1037/0022-3514.75.6.1559 9914666

[30] Robotham D Julian C. Stress and the higher education student: A critical review of the literature. J Further High Educ. (2006) 30:107–17. doi: 10.1080/03098770600617513

[31] Diez-Quevedo C Rangil T Sanchez-Planell L Kroenke K Spitzer RL. Validation and utility of the patient health questionnaire in diagnosing mental disorders in 1003 general hospital spanish inpatients. Psychosom Med. (2001) 686(63):679–86. doi: 10.1097/00006842-200107000-00021 11485122

[32] Kroenke K Spitzer RL. The PHQ-9: A new depression measure. Psychiatr Ann. (2002) 32:509–15. doi: 10.3928/0048-5713-20020901-06

[33] Spitzer RL Kroenke K Williams JBW Löwe B. A brief measure for assessing generalized anxiety disorder: The GAD-7. Arch Internal Med. (2006) 166(10):1092–7. doi: 10.1001/archinte.166.10.1092 16717171

[34] Garcia-Campayo J Zamorano E Ruiz MA Pardo A Perez-Paramo M Lopez-Gomez V. Cultural adaptation into Spanish of the generalized anxiety disorder-7 (GAD-7) scale as a screening tool. Health Qual Life Outcomes. (2010) 8(1):8. doi: 10.1186/1477-7525-8-8 20089179 PMC2831043

[35] Everaert J Tierens M Uzieblo K Koster EHW. The indirect effect of attention bias on memory via interpretation bias: Evidence for the combined cognitive bias hypothesis in subclinical depression. Cogn Emotion. (2013) 27(8):1450–9. doi: 10.1080/02699931.2013.787972 23627259

[36] Everaert J Duyck W Koster EHW. Attention, interpretation, and memory biases in subclinical depression: A proof-of-principle test of the combined cognitive biases hypothesis. Emotion. (2014) 14:331–40. doi: 10.1037/a0035250 24512247

[37] Sanchez A Duque A Romero N Vazquez C. Disentangling the interplay among cognitive biases: evidence of combined effects of attention, interpretation and autobiographical memory in depression. Cogn Ther Res. (2017) 41(6):829–41. doi: 10.1007/s10608-017-9858-5

[38] Cronbach LJ Furby L. How we should measure “change”: Or should we? psychol Bull. (1970) 74:68–80. doi: 10.1037/h0029382

[39] Benfer N Bardeen JR Clauss K. Experimental manipulation of emotion regulation self-efficacy: Effects on emotion regulation ability, perceived effort in the service of regulation, and affective reactivity. J Contextual Behav Sci. (2018) 10:108–14. doi: 10.1016/j.jcbs.2018.09.006

[40] Sanchez-Lopez A Everaert J Van Put J De Raedt R Koster EHW. Eye-gaze contingent attention training (ECAT): Examining the causal role of attention regulation in reappraisal and rumination. Biol Psychol. (2019) 142:116–25. doi: 10.1016/j.biopsycho.2019.01.017 30735680

[41] Stevens EN. The interactive effect of individual self-discrepancies: examining negative affective outcomes. J Soc Clin Psychol. (2015) 34(2):161–80. doi: 10.1521/jscp.2015.34.2.161

[42] Dickson JM Moberly NJ Huntley CD. Rumination selectively mediates the association between actual-ideal (but not actual-ought) self-discrepancy and anxious and depressive symptoms. Pers Individ Dif. (2019) 149:94–9. doi: 10.1016/j.paid.2019.05.047

[43] Gürcan-Yıldırım D Gençöz T. The association of self-discrepancy with depression and anxiety: Moderator roles of emotion regulation and resilience. Curr Psychol. (2022) 41:1821–34. doi: 10.1007/s12144-020-00701-8

[44] Liw L Han SY. Coping as a moderator of self-discrepancies and psychological distress. Couns Psychol Q. (2020) 35:284–302. doi: 10.1080/09515070.2020.1760208

[45] Scott L O’Hara MW. Self-discrepancies in clinically anxious and depressed university students. J Abnormal Psychol. (1993) 102:282–7. doi: 10.1037/0021-843X.102.2.282 8315140

[46] Hardin EE Lakin JL. The integrated self-discrepancy index: A reliable and valid measure of self-discrepancies. J Pers Assess. (2009) 91:245–53. doi: 10.1080/00223890902794291 19365765

[47] Johns A Peters L. Self-discrepancies and the situational domains of social phobia. Behav Change. (2012) 29:109–25. doi: 10.1017/bec.2012.1

[48] Ozgul S Heubeck B Ward J Wilkinson R. Self-discrepancies: measurement and relation to various negative affective states. Aust J Psychol. (2003) 55(1):56–62. doi: 10.1080/00049530412331312884

[49] Mason TB Smith KE Engwall A Lass A Mead M Sorby M. Self-discrepancy theory as a transdiagnostic framework: A meta-analysis of self-discrepancy and psychopathology. psychol Bull. (2019) 145(4):372–89. doi: 10.1037/bul0000186 30640499

[50] Brockmeyer T Anderle A Schmidt H Febry S Wünsch-Leiteritz W Leiteritz A. Body image related negative interpretation bias in anorexia nervosa. Behav Res Ther. (2018) 104:69–73. doi: 10.1016/j.brat.2018.03.003 29567546

[51] Henricks LA Lange W-G Luijten M van den Berg YHM Stoltz SEMJ Cillessen AHN. The longitudinal interplay between attention bias and interpretation bias in social anxiety in adolescents. Cogn Ther Res. (2022) 46:940–55. doi: 10.1007/s10608-022-10304-1

[52] Prieto-Fidalgo Á Calvete E. Bidirectional relationships between interpretation biases, safety behaviors, and social anxiety. Curr Psychol. (2023) 43:2597–606. doi: 10.1007/s12144-023-04461-z

[53] Feng Y-C Krahé C Koster EGW Lau JYF Hirsch CR. Cognitive processes predict worry and anxiety under different stressful situations. Behav Res Ther. (2022) 157:104168. doi: 10.1016/j.brat.2022.104168 35964460

[54] Blanco I Boemo T Martin-Garcia O Koster EHW De Raedt R Sanchez-Lopez A. Online Contingent Attention Training (OCAT): transfer effects to cognitive biases, rumination, and anxiety symptoms from two proof-of-principle studies. Cogn Res: Princ Implic. (2023) 8(1):28. doi: 10.1186/s41235-023-00480-3 37156967 PMC10166036

