# Supplementary material for: Do we interpret ambiguity and feel according to how we define ourselves? Relationships between self-perception, interpretation biases, and their role on emotional symptoms
Source: Front Psychiatry. 2024 Dec 20;15:1502130. doi: 10.3389/fpsyt.2024.1502130 (PMC11695330; doi:10.3389/fpsyt.2024.1502130)
Supplement: Supplementary file 1 [file Table1.docx]

**1. Supplementary Materials**

**1.1. Table 1.**

*Table 1:* Descriptives from variables included in the study.

|  | Temporal assessment | Mean (SD) | Internal consistency (Cronbach’s alpha) |
| --- | --- | --- | --- |
| Actual-Ideal SD (ranged from 0 to 6) | T1 | 1.49 (0.42) | .76 |
| Actual-Ought SD (ranged from 0 to 6) | T1 | 1.67 (.5) | .80 |
| Negative Interpretation Biases (ranged from 0 to 1) | T1 | .26 (.21) |  |
| Depressive symptoms (PHQ-9) (ranged from 0 to 27) | T1 | 7.62 (5.39) | .84 |
|  | T2 | 8.44 (5.77) | .88 |
| Anxiety symptoms (GAD-7) (ranged from 0 to 21) | T1 | 7.03 (4.97) | .89 |
|  | T2 | 7.6 (5.21) | .89 |

**1.2. Table 2.**

*Table 2:* Bivariate correlations from variables included in the study.

|  | Interpretation Bias | PHQ-9 | GAD-7 | Standardized Residuals PHQ-9 | Standardized Residuals GAD-7 |
| --- | --- | --- | --- | --- | --- |
| Actual-Ideal SD | .56*** | .51*** | .56*** | -.06 | -.07 |
| Actual-Ought SD | .54*** | .53*** | .52*** | -.04 | -.02 |
| Interpretation Bias | --- | .65*** | .56*** | -.05 | .14 |

*Note:* ***: p ≤ .001
